# Outcome registry of early intensive neurorehabilitation in patients with disorders of consciousness: study protocol of a prospective cohort study

**DOI:** 10.1186/s12883-021-02099-7

**Published:** 2021-02-12

**Authors:** Danielle M. F. Driessen, Cecile M. A. Utens, Gerard M. Ribbers, Willemijn S. van Erp, Majanka H. Heijenbrok-Kal

**Affiliations:** 1 Department of Rehabilitation Medicine, Erasmus MC, University Medical Centre Rotterdam, PO Box 2040, 3000 CA Rotterdam, the Netherlands; 2 Libra Rehabilitation & Audiology, PO Box 1355, 5022 KE Tilburg, the Netherlands; 3 Rijndam Rehabilitation, PO Box 23181, 3001 KD Rotterdam, the Netherlands; 4 Radboud University Medical Centre, Radboud Institute for Health Sciences, Department of Primary and Community Care, Nijmegen, the Netherlands; 5 Accolade Zorg, Zeist, the Netherlands

**Keywords:** Disorders of consciousness, Brain injury, Outcomes, Neurorehabilitation

## Abstract

**Background:**

Prolonged disorders of consciousness (PDOC) may occur after severe brain injury. Two diagnostic entities are distinguished within PDOC: unresponsive wakefulness syndrome (UWS, previously known as vegetative state) and minimally conscious state (MCS). Patients with PDOC may benefit from early intensive neurorehabilitation (EIN). In the Netherlands, the EIN programme is provided by one designated expert rehabilitation centre and forms the starting point of a dedicated chain of specialised rehabilitation and care for this group. This study project, called DOCTOR: Disorders of Consciousness; Treatment and Outcomes Registry, sets up a registry and systematically investigates multiple short- and long-term outcomes of patients with PDOC who receive EIN.

**Methods:**

Single-centre prospective cohort study with a 2-year follow-up period. Patients with PDOC due to acute brain injury who receive EIN, aged 16 years and older are included. Measurements will take place at start EIN, in week 5, 10, and at discharge from the EIN programme (duration = max 14 weeks) and at week 28, 40, 52, and 104 after admission to the EIN programme, following patients through the health-care chain. Outcome measures are the changes over time in level of consciousness, using the Coma Recovery Scale-Revised; the frequency and type of medical complications; the mortality rate; level of disability, including the level of motor, cognitive, behavioural and emotional functioning; participation; and quality of life. Secondary outcomes include self-efficacy of caregivers, caregivers’ strain and cost-effectiveness of the programme.

**Discussion:**

The DOCTOR study will provide insight in the recovery patterns and predictors of recovery for multiple outcomes in PDOC patients after following EIN. The results of the study will enable us to benchmark and improve EIN and the organisation of the health-care chain, both for patients with PDOC and for their families.

**Trial registration:**

Netherlands Trial Register, NL 8138. Retrospectively registered 6 November 2019.

## Background

Prolonged disorders of consciousness (PDOC) (> 4 weeks) due to acquired brain injury (ABI) although uncommon [1, 2] have a major impact on patients and their families [3]. Two diagnostic entities are distinguished within PDOC: the unresponsive wakefulness syndrome (UWS, previously known as vegetative state) and the minimally conscious state (MCS) [4, 5]. In UWS, persons display spontaneous eye opening and sleep/wake cycles, but observable signs of purposeful behaviour (e.g. language comprehension, behavioural response to stimuli) are absent [6]. Patients in MCS demonstrate minimal but definitive behavioural evidence of awareness. Conscious behaviour in MCS is often subtle and inconsistent, and must be systematically differentiated from reflexive or random behaviour [6]. Based on the complexity of behaviour, a differentiation is made between MCS-minus (MCS-) and MCS-plus (MCS+) [7, 8]. MCS+ is distinguished from MCS- by the evidence of language processing [7]. PDOC can arise from various aetiologies, but the most common causes in adults are traumatic brain injury, intracerebral and subarachnoid haemorrhage and hypoxic-ischemic encephalopathy [4, 9, 10]. Hypoxic-ischemic encephalopathy is the most common cause in UWS patients (38–50%) [9, 11], while cerebral haemorrhage is most common in MCS [9]. Van Erp et al. reported a prevalence of 0.1 to 0.2 hospitalised and institutionalised UWS patients per 100,000 members of the general Dutch population [11] while worldwide prevalence varies from 0.2 up to 6.1 per 100,000 [1]. PDOC patients may benefit from subacute neurorehabilitation, such as early intensive neurorehabilitation (EIN). EIN focuses on (1) basic care and prevention of complications [12] (e.g. thrombo-embolic disease, contractures, skin breakdown, paroxysmal sympathetic hyperactivity), which cause a substantial raise in morbidity, discomfort and pain, and in healthcare costs [13]; (2) accurate assessment and treatment aimed to facilitate recovery of level of consciousness (LOC); (3) finally, providing comprehensive education and empowerment of families to foster families’ coping.

Research on recovery shows that 38 and 78% of patients with PDOC due to traumatic brain injury (TBI) regain (minimal) consciousness at respectively 3 and 12 months post injury [2]. In patients with non-traumatic brain injury (NTBI) only 17% regains consciousness at 8 months post injury [2]. Aidinoff et al. [14] demonstrated an improvement in survival and recovery of consciousness in UWS patients over the last two decades after EIN, and similar outcomes for both TBI and NTBI UWS. These outcomes suggest that improvements in acute medical care and EIN have contributed to advances in UWS care. Treatment started within 8 weeks after injury is associated with better outcomes in terms of LOC and functioning, gained in a faster pace than treatment started after 8 weeks [15–17]. In patients with PDOC of mixed aetiology with a mean time follow-up of 5 years, 40% of survivors reached functional independence, while 42% of patients able to respond to a self-report questionnaire, reported a poor quality of life [18]. The EIN programme in the Netherlands has been studied before; in patients aged < 25 years recovery of consciousness occurred in two-thirds at the end of the EIN programme [19] and two-thirds of the patients who were fully conscious at discharge were able to live independently after 10–12 years [20].

Prognostic factors associated with better recovery from PDOC are younger age at onset, traumatic aetiology, less severe comorbidities and less severe neurological impairment, i.e. higher level of consciousness at admission to EIN [2, 4, 15, 21–25]. However, most studies on recovery after PDOC suffer from methodological issues, such as a retrospective design [2] and variability in clinical decision making in acute care [2]. Further, research on outcomes after PDOC often fails to discriminate between diagnostic subtypes (UWS or MCS), aetiology and the time since injury [2]. Furthermore, data on quality of life, costs and cost-effectiveness of rehabilitation programmes, and caregiver burden are limited.

The **D**isorders **o**f **C**onsciousness **T**reatment and **O**utcomes **R**egistry (DOCTOR) is a prospective follow-up study on outcomes for all patients ≥16 years in UWS and MCS and their families who entered the EIN programme in one designated expert rehabilitation centre in the Netherlands. It is a 14-week subacute intensive neurorehabilitation programme for patients discharged from the intensive care unit or hospital ward, to improve (recovery of) consciousness, prevent complications and empower caregivers.

In DOCTOR outcomes of patients with PDOC are recorded systematically with a follow-up of 24 months after admission to EIN. Main outcome measures are (predictors) of recovery of LOC, mortality, incidence and type of complications, outcomes in terms of level of disability, including the level of motor, cognitive, behavioural and emotional functioning, pain, cognition, participation and quality of life. Further, caregiver burden and self-efficacy will be assessed as well as cost-effectiveness.

## Methods

### Study design and setting

**Fig. 1:**
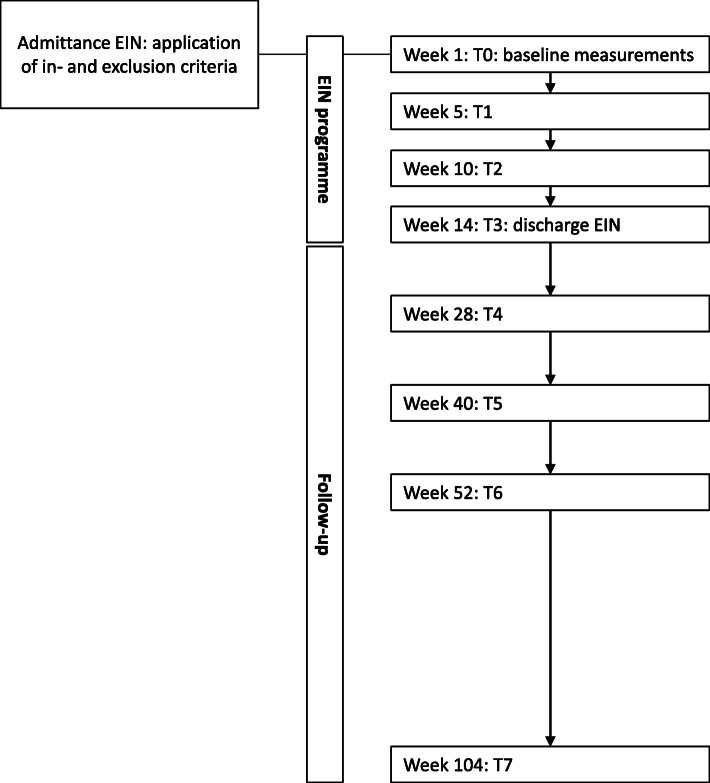
Study design

The DOCTOR study is a nationwide single-centre prospective cohort study with 2 years follow-up. Measurements take place at start EIN, in week 5, 10, and at discharge of the EIN programme (duration = max 14 weeks) and at week 28, 40, 52, and 104 after admission to the EIN programme, following patients through the health-care chain. (Fig. 1).

The EIN programme is standard care and is provided in a single specialised rehabilitation centre in the Netherlands. All hospitals in the Netherlands may refer patients who are eligible for EIN to this centre. Informed consent is obtained from the legal representative of each patient before the start of the study. Patients who show sufficient recovery of consciousness during the study trajectory will personally be asked consent for further participation in the study.

This study is not subject to the “Medical Research Involving Human Subjects Act”, determined by the Medical Ethics Committee of Erasmus MC, University Medical Centre Rotterdam (MEC-2019-0127).

### In- and exclusion criteria

Patients with PDOC due to acute brain injury who are admitted to the EIN programme are possible candidates for this study. At admission to EIN, patients will be screened for eligibility for DOCTOR. Patients are included in DOCTOR if the in- and exclusion criteria are met (Table 1).

**Table 1:** Inclusion and exclusion criteria

**Inclusion criteria**	
Age16 years or older	
PDOC (UWS or MCS) lasting > 4 weeks and < 6 months at admission to EIN	
First-time newly acquired non-progressive brain injury of any aetiology	
Weaned from ventilator	
Medically stable, as judged by the treating rehabilitation physician	
**Exclusion criteria**	
Coma	
Any pre-existent progressive or non-progressive brain injury	
Uncontrollable epilepsy	

### EIN programme

The EIN programme is a subacute medical rehabilitation programme of 14 weeks (or less when recovery of consciousness occurs earlier) and meets the guideline recommendations for patients with PDOC published by the American Academy of Neurology [26]. An interdisciplinary rehabilitation team of a rehabilitation physician, a physician assistant, a neuropsychologist, cognitive rehabilitation therapists, physiotherapists, occupational therapists, speech therapists, nursing staff, dietician, and social worker uses an integrated approach in a 24/7 setting [19] with a minimum duration of 120 min therapy per day in which daily routines are built in together with a structured scheme of therapy and rest. The program is focused on optimizing the metabolic state, nourishment, respiration and skin condition, as well as diminishing the risk of infections and quick removal of invasive devices, like a tracheostomy tube or a urinary catheter, and recovery of the normal circadian cycle. Pharmacological treatments and structured stimulation of all sensory modalities (vision, hearing, smell, taste, touch, posture and motion, pain and temperature) aim to generate maximal arousal. As soon as the patient shows any voluntary reactions, reflecting a change from UWS into MCS, the focus of treatment changes from stimulation to training of cognitive functions. Motor rehabilitation aimed at optimising postural and motor activity helps restore bodily integrity and reduces the risk of complications (e.g., infection, thrombo-embolic complications, skin breakdown). For patients who reach MCS, increased time is spent on training mobility and self-care. Coping and self-efficacy of caregivers are addressed with psychoeducation, counselling and hands-on training in handling the patient.

**Fig. 2:**
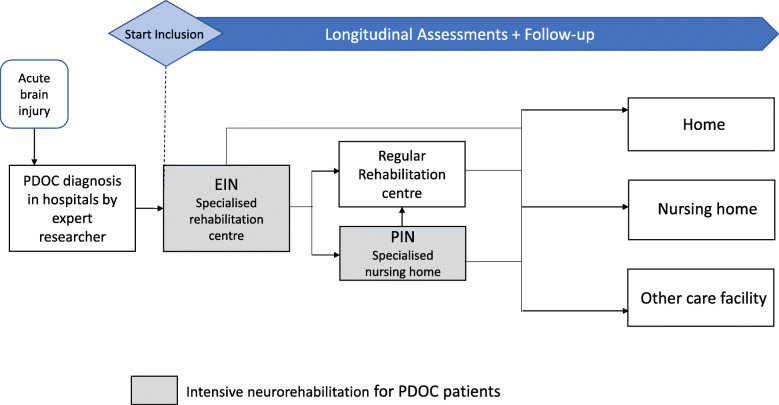
Healthcare pathways for PDOC. EIN: Early intensive neurorehabilitation –duration max 14 weeks. PIN: Prolonged intensive neurorehabilitation –up to 2 years post injury

After EIN, patients are discharged to 1) a regular neurorehabilitation setting in case of full recovery of consciousness and the ability to participate in a rehabilitation programme as judged by the rehabilitation physician, 2) to skilled nursing facilities, 3) sheltered housing or 4) possibly home (Fig. 2). Some skilled nursing facilities offer a prolonged intensive neurorehabilitation programme (PIN) for further recovery of consciousness during a period of up to 24 months post-injury. EIN is part of an academic network of expertise (EENnaComa) in which various healthcare institutes specialised in post-acute and long-term care of PDOC patients and their families are united.

**Fig. 3:**
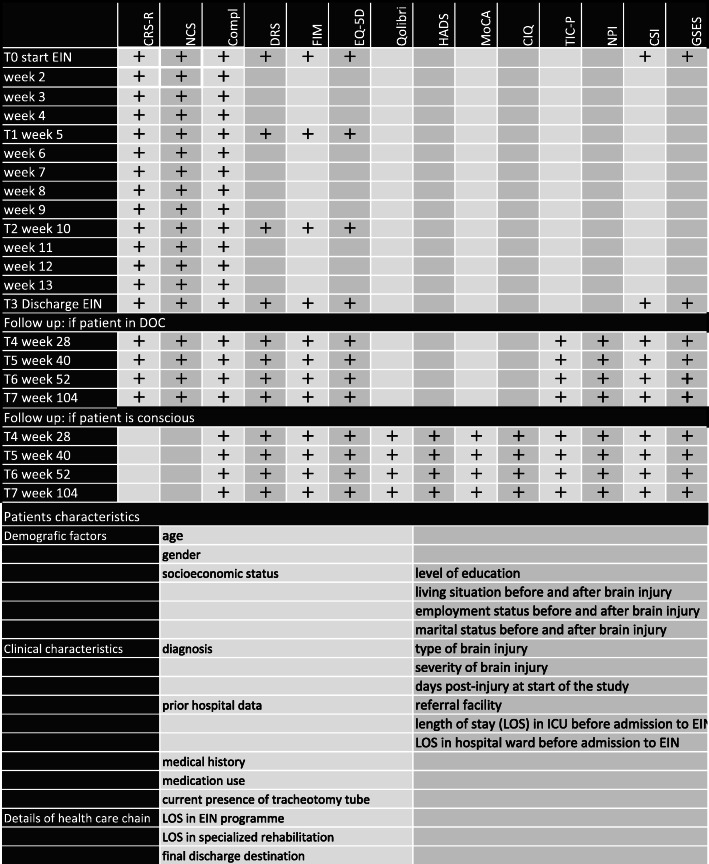
Measurement plan

### Data collection

In 4 years we aim to include 72 patients. Structured data collection (Fig. 3) will take place at the start of the EIN programme at week 5, 10, and at EIN discharge (week 14). LOC assessment and registration of complications will be done weekly.

After discharge from EIN, follow-up measurements are planned at 28, 40, 52, and 104 weeks post-inclusion. These will take place at the patients’ actual health-care institution or residence.

All measurements conducted during the EIN programme are performed by trained members of the rehabilitation team. Follow-up measurements are performed by a trained research assistant.

### Complications

The number and type of complications is classified according to the International Classification of Functioning, Disability and Health (ICF) in the domain of functions and structures (Table 2). In addition, the number of imaging studies and types of treatments for complications (surgery, non-surgery, ICU transfers, procedures i.e.) are recorded.

**Table 2:** Classification of complications

• Mental/nervous system	
o Epilepsy	
o Hydrocephalus	
o Paroxysmal Sympathetic Hyperactivity (PSH)	
o Disturbed sleep/wake cycles	
• Sensory systems	
• Cardiovascular, haematologic, immunologic and respiratory systems	
o Thromboembolic disease	
o Abnormal laboratory findings (e.g. hyponatremia)	
• Digestive, metabolic and endocrine systems	
o Gastro-intestinal: vomiting, diarrhoea, obstipation	
• Genitourinary and reproductive systems	
• Musculoskeletal and movement-related systems	
o Contractures measured by joint range of motion (ROM)	
o Spasticity: Modified Ashworth scale	
o Heterotopic ossification	
• Skin and related structures	
o Skin breakdown: EPUAP stages I-IV (Dutch guideline: pressure ulcer: prevention and treatment [27]	
• Infection	
Body system	
o Respiratory	
o Urinary tract	
o Blood	
o Stool	
o CSF, brain	
o Wound/skin	
o Ear/eye	
Antibiotic-resistant bacteria	

### Mortality

We will register the following causes of death: [28].

- Death from (co) morbidity despite treatment
- Death from comorbidity after a non-treatment decision (non-resuscitation or withholding treatment for medical complications)
- Death after withdrawal of artificial nutrition and hydration.

### Measurement instruments

#### Coma recovery scale revised (CRS-R)

The CRS-R [29] is a bedside assessment tool for differentiating levels of consciousness by observation of responses to various stimuli. It is composed of 6 hierarchical subscales: auditory, visual, motor, oromotor/verbal, communication and arousal with 23 dichotomously scored items [30]. The item content of the CRS-R identifies ascending levels of conscious behaviour/ability and using a combination of items to differentiate UWS from MCS from emerged from an MCS based on The Multi-Society Task Force on Persistent Vegetative State and Aspen Workgroup criteria [29]. An American Congress of Rehabilitation Medicine practice parameter recommends the use of the CRS-R based on its standardised administration and scoring procedures, item content, and inter-rater and test-retest reliability [26, 29].

#### Nociception coma scale (NCS)

The NCS is a nociception behaviour observation tool, developed for patients with PDOC [31]. It includes four subscales assessing motor, verbal, visual and facial responses; each subscore ranges from 0 to 3 (total score: 0–12). A higher total score indicates a higher level of possible discomfort and pain [32].

#### Disability rating scale (DRS)

The DRS is an observational measurement of the levels of arousal, awareness, and responsiveness (including eye opening, communication ability, and motor response); cognitive ability of self-care activities (such as feeding, using the toilet, and grooming); dependency on others (level of functionality); and psychosocial adaptability (employability). The DRS score ranges from 0 to 29 (low to high level disability) and scores are categorised into the following disability categories: 0 = none, 1 = mild, 2–3 = partial, 4–6 = moderate, 7–11 = moderately severe, 12–16 = severe, 17–21 = extremely severe, 22–24 = vegetative state, and 25–29 = extreme vegetative state. The DRS has been used as an outcome measure in multiple previous studies on PDOC outcome [13, 15, 17, 19].

#### Functional Independence measure (FIM)

The FIM measures the level of a patient’s disability and indicates how much assistance is required for conducting activities of daily living [33]. The FIM contains 18 items composed of 13 motor tasks and 5 cognitive tasks. Tasks are rated on a 7-point scale, ranging from 1 = total assistance to 7 = complete independence. FIM total scores range from 18 to 126, with higher scores reflecting a higher level of functioning [34].

#### Community integration questionnaire (CIQ)

The CIQ is a 15-item questionnaire that assesses participation in 3 domains: within the home environment, in social interactions, and in productive activities (work, school, or volunteering) [35]. The basis for scoring is primarily the frequency of performing activities or roles, with secondary weight given to whether activities are done jointly with others, and the nature of these other persons (for example, with/without TBI). CIQ scores range from 0 to 29, a higher total score indicates a higher level of participation.

#### EuroQuol-5D-5L (EQ-5D-5L)

Health-related quality of life is measured using the EQ-5D-5L [36, 37]. It consists of a 5-item EQ-5D index (mobility, self-care, usual activities, pain/dis- comfort and anxiety/depression) and a visual analogue scale (EQ-VAS). Psychometric properties of the Dutch version are shown to be good. The five EQ-5D index items are summarised into one weighted overall score, which runs from 0 for the value of death to 1.00 for full health. The EQ-VAS ranges from 0 to 100 (worst to best imaginable health state). For the general Dutch population average EQ-5D index = 0.843 and average EQ-VAS = 81.36 [37]. At the start of the study, the questionnaire will be completed only by proxy, as soon as the patient regains consciousness, the questionnaire will be filled in both by proxy and patient.

#### Quality of life in brain injury (QOLIBRI)

The QOLIBRI was developed by an international task force in two multi-language studies involving more than 2000 persons after traumatic brain injury (TBI) [38]. The QOLIBRI is a comprehensive questionnaire with 37 items covering six dimensions of disease-specific health-related quality of life (HRQoL). The questionnaire provides a profile of quality of life together with a total score. The QOLIBRI scores are reported on a 0–100 scale, where 0 is the worst possible quality of life and 100 the best possible quality of life.

#### Hospital anxiety and depression scale (HADS)

The Dutch Hospital Anxiety and Depression Scale is used as a general measure of emotional distress and contains two subscales: anxiety and depression [39]. Subscale scores ≥8 might indicate the presence of a depressive disorder or an anxiety disorder. Reliability and validity are adequate for several clinical populations, including multiple sclerosis and acquired brain injury [40, 41].

#### The Montreal cognitive assessment (MoCA)

The MoCA is designed as a rapid screening instrument for cognitive dysfunction [42]. It assesses different cognitive domains: attention and concentration, executive functions, memory, language, visioconstructional skills, conceptual thinking, calculations, and orientation. The total possible score is 30 points; a score of ≥26 is considered as normal, 18–25 indicates mild cognitive impairment, 10–17 moderate cognitive impairment and < 10 severe cognitive impairment.

#### Neuropsychiatric inventory (NPI)

The Neuropsychiatric Inventory (NPI) is designed to be a retrospective (last month) interview assessing neuropsychiatric symptoms conducted with informants about patients for whom they care. The NPI consists of 12 domains, with each domain reflecting a cardinal neuropsychiatric symptom. The NPI provides symptom frequency, severity and distress ratings for each symptom reported, and total severity and frequency scores reflecting the sum of individual domain scores (range 0–144). A higher total score indicates a higher number of neuropsychiatric symptoms [43, 44].

#### Self-efficacy of caregiver (GSES)

Partners’ general self-efficacy is measured using the General Self-efficacy scale (GSES) [45]. This self-assessment scale contains 10 items scored on a 4-point scale ranging from ‘completely incorrect’ to ‘completely correct’. By adding all item scores, a total score is obtained with higher scores indicating greater self-efficacy.

#### Caregiver strain index (CSI)

The CSI assesses informal caregiver’s burden. The CSI contains 13 items. Scores range from 0 to 13, with higher scores indicating higher burden [46].

#### Trimbos and iMTA questionnaire on costs associated with psychiatric illness (TiC-P)

The TiC-P will be used to collect data on healthcare use from both a healthcare and societal perspective during follow-up. This questionnaire consists of two parts. The first part consists of questions on the number of consultations with healthcare providers during the previous 2 months. These include outpatient visits, hospital admissions, day care, etc. Also, the frequency and dosage of medication use is recorded. The second part of the TiC-P includes the Short Form of the Health and Labour Questionnaire (SF-HLQ), which involves questions on absence from and return to paid or voluntary employment for measuring productivity losses [47].

### Statistical analysis

Descriptive statistics will be used to present the outcomes of the study parameters and the socio-demographic data. Means and standard deviations will be calculated for variables on an interval scale and medians and interquartile ranges for ordinal variables. Proportions will be calculated for nominal variables.

Subgroup analyses will be performed for patients presenting in UWS versus MCS at baseline and for traumatic versus non-traumatic disorders of consciousness.

The proportions of patients transitioning from UWS to MCS and emergence from MCS to the conscious state will be calculated during and after EIN.

The course of recovery of consciousness (yes/no) over time will be analysed using Generalized Estimating Equations (GEE) analysis for logistic outcomes on an intention-to-treat basis. Assuming that data are missing at random, data imputation is unnecessary in GEE analysis; all observations are taken into account in contrast to complete case analyses. Recovery (yes/no) at each time point will be included as the dependent variable in the GEE model. Separate models will be generated for transitions from UWS to MCS and from MCS to the fully conscious state based on the CRS-R. Independent variables that will be added one by one to the models, include- measurement time (weeks), traumatic vs. non-traumatic aetiology, age at injury, and complications (yes/no or number). Significant predictors (*p* < 0.05) will be included in a multivariable model using a Bonferonni correction, dividing the significance level (alpha< 0.05) by the number of predictors included in the model.

Also using GEE analysis, the course of quality of life (EQ-5D-5L and Qolibri) and participation (CIQ) over time will be analysed using separate linear outcome models. The effects of time, cognitive (MoCa), behavioural (NPI), emotional (HADS), and motor status (FIM), and level of disability (DRS) and discomfort (NCS) on quality of life or participation during follow-up will be studied by adding these time-dependent variables (one by one) as independent variables to the models. Significant predictors (*p* < 0.05) will be added into a multivariable GEE-model to study the independence of these potential predictors on the outcomes using the Bonferroni correction.

In caregivers, self-efficacy (GSES) and caregiver strain (CSI) are measured, which will also be modelled using GEE analyses. Independent variables added to these models include age and gender of the caregiver and the level of consciousness (UWS, MCS, or conscious), and the cognitive (MoCA) and motor (FIM) status of the patients.

## Discussion

In this manuscript we present the DOCTOR study on outcomes of EIN in patients with PDOC due to acute brain injury. EIN is open to the entire adult Dutch PDOC population centralised in a single specialised centre. This study complies with recent guidelines on PDOC of The Royal College of Physicians (2020), which advocates the establishment of a national registry and agreed on a minimum dataset for the collection of national cohort of longitudinal outcome data for all patients in PDOC [48].

Besides clinical characteristics, functional outcomes and employment, caregiver’s burden and the quality of life in both patient and their family will be assessed. The results of the study will provide insight in the recovery patterns and the predictors of recovery of PDOC in the Netherlands, which will enable us to benchmark and improve EIN and to improve the prediction of outcome.

Embedded in the DOCTOR study is the TOPDOC study. The TOPDOC study is a qualitative study that focuses on clinical decision-making regarding treatment and end-of-life care, ethical dilemmas, quality of outcomes, dying and quality of dying in PDOC, involving patients, families and healthcare professionals. The DOCTOR and TOPDOC studies provide a rich and solid dataset that will grasp the complexity of PDOC in terms of functional and meaningful recovery and gives a unique opportunity to improve and strengthen integrated care pathways for PDOC care in the Netherlands.

There are some limitations in this study that need to be discussed. First, as EIN is standard care a randomized controlled trial design is not feasible for ethical reasons. Second, LOC determination in PDOC is difficult in the absence of a gold standard. Recent guidelines advocate the use of additional diagnostic techniques such as EEG beside a behavioural assessment in the diagnostic process [26, 49]. However, to date these are not implemented in daily PDOC care in the Netherlands. In DOCTOR, we use the CRS-R, the most frequently used instrument worldwide [26]. While assessing LOC by using CRS-R, confounding factors such as motor, visual, auditory and/or cognitive impairments (e.g., language, memory, flexibility, attention) [50], intubation, sedation and the setting (e.g., presence or absence of relatives) should be taken into consideration [51], as they may negatively influence the diagnostic evaluation. Third, the follow-up time of our study is limited to 2 years. While some patients may show later recovery, the most significant proportion of recovery of consciousness after brain injury takes place in the first 1 to 1.5 years [48]. However, due to the high rate of misdiagnosis in PDOC, the evidence on ‘late’ recovery from PDOC may suffer from a diagnostic delay bias [52]. While most functional recovery occurs by 1-year post-injury, improvements may occur over a more prolonged interval. Some studies have shown significant improvement in cognitive and motor function in TBI patients admitted to inpatient rehabilitation from rehabilitation discharge to year 1, year 1 to 2, year 2 to 5 and year 5 to 10. However, the gains noted across functional areas by 2, 5 and 10 years after injury were relatively small compared with the first year post-injury [17, 53, 54]. Therefore, we think that a follow-up of 2 years will provide a good representation of the recovery after DOC in the long term, both for regaining consciousness and level of cognitive, behavioural, emotional and motor functioning.

To summarize, in this contribution we present the protocol of the DOCTOR study: a nationwide single-centre prospective cohort study of the outcomes of PDOC patients enrolled in Early Intensive Neurorehabilitation, followed up to 2 years after admission, which will provide insight in the long-term recovery patterns and predictors of multiple outcomes in patients with PDOC.

## Data Availability

Not applicable.

## Abbreviations

ABI: Acquired Brain Injury
CRS-R: Coma Recovery Scale-Revised
DOC: Disorders of Consciousness
EIN: Early Intensive Neurorehabilitation
LOC: Level of Consciousness
LOS: Length of Stay
MCS: Minimal Conscious State
NTBI: Non-Traumatic Brain Injury
PDOC: Prolonged Disorders of Consciousness
UWS: Unresponsive Wakefulness Syndrome
TBI: Traumatic Brain Injury
VS: Vegetative State
